# Syntaxin 8 Regulates Platelet Dense Granule Secretion, Aggregation, and Thrombus Stability*[Fn FN2]

**DOI:** 10.1074/jbc.M114.602615

**Published:** 2014-11-17

**Authors:** Ewelina M. Golebiewska, Matthew T. Harper, Christopher M. Williams, Joshua S. Savage, Robert Goggs, Gabriele Fischer von Mollard, Alastair W. Poole

**Affiliations:** From the ‡ School of Physiology and Pharmacology, Medical Sciences Building, University Walk, Bristol BS8 1TD, United Kingdom,; the § School of Cancer Sciences, University of Birmingham, Edgbaston, Birmingham B15 2TT, United Kingdom,; the ¶ Department of Clinical Sciences, College of Veterinary Medicine, Cornell University, Ithaca, New York 14853, and; the ‖ Fakultät für Chemie, Biochemie III, Universität Bielefeld, Postfach 100131, 33501 Bielefeld, Germany

**Keywords:** ADP, Exocytosis, Platelet, SNARE Proteins, Thrombosis

## Abstract

Platelet secretion not only drives thrombosis and hemostasis, but also mediates a variety of other physiological and pathological processes. The ubiquitous SNARE machinery and a number of accessory proteins have been implicated in regulating secretion in platelet. Although several platelet SNAREs have been identified, further members of the SNARE family may be needed to fine-tune platelet secretion. In this study we identified expression of the t-SNARE syntaxin 8 (STX8) (Qc SNARE) in mouse and human platelets. In mouse studies, whereas STX8 was not essential for α-granule or lysosome secretion, *Stx8*^−/−^ platelets showed a significant defect in dense granule secretion in response to thrombin and CRP. This was most pronounced at intermediate concentrations of agonists. They also showed an aggregation defect that could be rescued with exogenous ADP and increased embolization in *Stx8*^−/−^ mice *in vivo* consistent with an important autocrine and paracrine role for ADP in aggregation and thrombus stabilization. STX8 therefore specifically contributes to dense granule secretion and represents another member of a growing family of genes that play distinct roles in regulating granule release from platelets and thus platelet function in thrombosis and hemostasis.

## Teaser

**Background:** The molecular machinery controlling exocytosis of the three secretable granules types in platelets is not fully elucidated.

**Results:** ATP secretion, aggregation, and thrombus stability are defective in *Stx8*^−/−^ mouse platelets.

**Conclusion:** STX8 is involved in platelet dense granule secretion, and the STX8-mediated pathway contributes to thrombus stabilization.

**Significance:** Identification of the novel functional SNARE STX8 suggests alternative mechanisms of granule secretion exist in platelets.

## Introduction

Platelet secretory granules, α-granules, dense granules, and lysosomes contain a plethora of active substances that regulate thrombosis and hemostasis, and increasingly found to contribute to other physiological and pathological processes, such as inflammation (1), angiogenesis (2), and malignancy (3).

Our understanding of platelet secretion has developed tremendously since the first description of the ubiquitous SNARE secretory machinery in platelets by Lemons *et al.* (4). We now know that regulatory proteins including small GTPases (5–7), MUNC proteins (8, 9), and calcium sensors (10, 11) contribute to regulation of platelet secretion alongside the “core” SNARE complex.

The functional core SNARE complex invariably consists of 4 SNARE domains: three t-SNARE domains (residing on the target membrane) and a v-SNARE on the vesicle form a tight heterotetrameric complex that drives membrane fusion (12). The majority of mammalian SNAREs, including all syntaxins and SNAP23 homologues, are classified as t-SNAREs, whereas VAMPs function as v-SNAREs. Although SNAREs are functionally classified as v-SNAREs or t-SNAREs, they can also be structurally distinguished as Qabc or R types according to the amino acid residue (glutamine and arginine) located in the center of the functional complex (13). Both the functional organization and the structure of the central SNARE complex are remarkably conserved between cells, and even species (14).

The currently accepted platelet secretion machinery is the complex of STX11^2^ (a Qa SNARE), SNAP23 (Qbc), and VAMP8 (an R SNARE); however, in light of the level of temporal and spatial regulation required in platelet secretion, the “one size fits all” model does not seem to be plausible. In addition, even loss of STX11 (in FHL4 patients) (15) or knock-out of VAMP8 coupled with tetanus neurotoxin (TnT-LC) treatment (16) does not lead to full ablation of secretion, suggesting ranked redundancy and compensation mechanisms (16). The evidence supporting SNAP23 is even more indirect and reliant upon treatment of SLO-permeabilized platelets with functional blocking antibodies and the correlative evidence of phosphorylation of SNAP23 occurring at a similar rate to secretion (17–20).

There are numerous examples of SNARE redundancy and specialization in secretory cells, for example, neutrophils (21). However, in those cells approaches such as protein overexpression or targeted introduction of specific antibodies by electropermeabilization (21) allow for easier characterization of SNARE function in secretion. Characterization of similar processes in platelets remains more challenging.

We hypothesized that other SNARE proteins may help “fine-tune” secretion alongside the VAMP8-STX11-SNAP23 complex. There is substantial expression evidence that additional SNAREs are present in platelets, but currently there are several of these that have not been investigated (22–25). We decided to prioritize characterization of a syntaxin STX8, an abundant Qc SNARE, which could partially substitute for SNAP23 (which in the current model contributes both Qb and Qc domains) in platelet SNARE complexes (26).

Here we report that syntaxin 8 (STX8) (Qc) is expressed in mouse and human platelets, and localizes to the membrane fraction of platelets. We have also found that it forms a complex with STX11 in human platelets, which unlike the activation-dependent STX11-SNAP23-VAMP8 complex, is present in resting platelets. We also show a novel role for STX8 in dense granule secretion in mouse platelets in response to intermediate agonist stimulation *in vitro*, and impairment of aggregation secondary to this defect. Most importantly, however, we show that the embolization rate from thrombi *in vivo* was significantly increased in the absence of STX8, whereas the total size of the thrombus and rate of adhesion as measured by traditional methods remained unchanged. This suggested that the relatively subtle deficit in dense granule secretion can result in a substantial physiological defect downstream.

This study is the first to suggest a role for STX8 in platelet secretion and function, which opens up the possibility of other SNAREs playing secondary roles to the “main” SNARE complex, as previously reported in the case of VAMP homologues (16). Our results are consistent with previous suggestions of differential secretion of granule types in platelets. In addition, we show that apparently minor defects in dense granule secretion observed *in vitro* can still have significant effects *in vivo*.

## EXPERIMENTAL PROCEDURES

### 

#### 

##### Human and Mouse Platelet Preparation

For human platelet experiments, blood was taken from aspirin-free healthy volunteers who gave full informed consent. For mouse platelet experiments, *Stx8*^−/−^ embryos were supplied by the Wellcome Trust Sanger Institute (Cambridge, UK), and rederived at the University of Bristol animal facility. *Vti1b*^−/−^ mice were generated as described previously (27). Mice, minimum 8 weeks of age, of mixed sex were used for all experiments, and compared with wild-type littermates or sex- and age-matched C57Bl6/j wild-type controls. Blood was drawn into 4% trisodium citrate (1:9) by posterior vena cava puncture of mice humanely euthanized by exposure to rising CO_2_ gas concentrations as per Schedule 1 of ASPA, 1986. Complete blood counts were conducted using a Pentra ES60 hematology analyzer (Horiba Medical, Northampton, UK) prior to platelet preparation and counts were adjusted for anticoagulant volume. Washed platelets were prepared as described in Goggs *et al.* (28), and rested for 60 min at 30 °C in the presence of 10 μm indomethacin and 0.02 units/ml of apyrase prior to stimulation. Experiments were performed in the presence of indomethacin as a standard to minimize signaling contributions from receptors other than the primary receptor being activated.

##### Protein Electrophoresis and Western Blotting

Washed platelet lysates were separated on SDS-PAGE, and PVDF membranes were probed with specific primary antibodies: VAMP8, SNAP23, STX11, VTI1B (all rabbit IgG, Synaptic Systems GmBH, Göttingen, Germany), STX8 (sheep IgG, R&D Systems, Abingdon, UK), GAPDH (mouse IgG, Santa Cruz Biotechnology, Heidelberg, Germany), α-tubulin (mouse IgG), and FcRγ (rabbit IgG, both Abcam, Cambridge, UK). Membranes were washed in excess TBS-T and incubated with appropriate HRP-conjugated secondary antibodies (mouse and rabbit, GE Healthcare Life Sciences; sheep, Jackson ImmunoResearch, Stratech, Newmarket, UK). Protein levels were quantified using ImageJ analysis software and compared using ANOVA.

##### Digitonin Fractionation of Human Platelets

Human platelets were incubated with an equal volume of 0.1% digitonin in phosphate-buffered saline (PBS) supplemented with protease inhibitor mixtures for 120 s on ice with intermittent agitation. The samples were centrifuged at 1,800 × *g* for 10 min at 4 °C to separate the soluble (cytosolic) fraction from the digitonin-insoluble membrane-bound fraction. The supernatants were extracted with 4× SDS sample buffer. The remaining insoluble pellet was washed twice with ice-cold PBS and solubilized with 0.1% Triton X-100 in PBS for 5 min and centrifuged as before. The supernatant was extracted as before and membrane and cytosol fractions were separated using SDS-PAGE, and localization was confirmed with Western blotting relative to FcRγ (membrane fraction marker) and GAPDH (cytosolic fraction marker).

##### Co-immunoprecipitation

Double-washed human platelets, resting or activated with 1 unit/ml of thrombin, were lysed in equal volumes of 2× ice-cold lysis buffer (40 mm Tris-HCl, pH 7.5, 150 mm NaCl, 2 mm EDTA, 2 mm EGTA, 2% Triton X-100, 1% sodium deoxycholate, protease and phosphatase inhibitors). Lysates were precleared by centrifugation at 4 °C for 15 min at 1800 × *g* and incubated with 2 μg of relevant primary antibody or isotype-nonspecific IgG overnight at 4 °C. Immune complexes were precipitated with Protein A or G beads blocked with 2% BSA protein eluted by boiling for 5 min at 95 °C. The eluates were then separated by electrophoresis as described.

##### Dense Granule Secretion and Platelet Aggregation

Aggregation and ATP secretion were measured under stirring (1000 RPM) conditions using Born lumi-aggregometer (560-VS, Chrono-Log, Havertown, PA) as previously described (28). Aggregation was expressed as a decrease in optical density of the sample. ATP secretion was normalized to WT control on each experimental day as appropriate.

##### Flow Cytometry

Expression of surface glycoproteins was measured by incubating platelets (2 × 10^7^/ml) with FITC-labeled antibodies for CD41, GPVI, and GPIbα or isotype-nonspecific IgG for 10 min (all Emfret, Germany). For surface expression of P-selectin and integrin α_IIb_β_3_ activation assay, platelets were stimulated with agonists under non-stirring conditions and incubated with FITC-CD62P and PE-JON/A antibodies (both Emfret) for 10 min. Platelets were analyzed using FACSCalibur and proprietary software (Cell Quest, BD Bioscience). Platelets were identified by forward and side-scatter properties. For dual color experiments, compensation controls were produced using FITC- and PE-stained beads (Calibrite Beads, BD Bioscience) and applied to all fluorescence intensity values. 20,000 platelet-gate events were collected per experiment.

##### Lysosome Secretion

β-Hexosaminidase release was measured as described previously (29). Mouse platelets were stimulated with increasing concentrations of thrombin at 37 °C for 10 min under non-stirring conditions and the supernatant added to 20 μl of 0.1 m 4-nitrophenyl *n*-acetyl-β-d-glucosaminide (Sigma) substrate in citrate-phosphate buffer (0.2 m Na_2_HPO_4_, 0.1 m citric acid, pH 4.2) in a 96-well plate. Total controls for β-hexosaminidase activity were obtained by repeated snap-freeze/thaw of the same volume of non-stimulated platelets. The reaction was quenched with NaOH after a 1-h incubation at 37 °C and absorbance was read at 405 nm (Opsys MR, Dynex Technologies, Worthing, UK). Secreted β-hexosaminidase was expressed as percentage of TOTAL.

##### [^3^H]5-HT Loading Assay

Mouse platelet-rich plasma was incubated with ^3^H-labeled 5-HT (PerkinElmer Life Sciences) (0.5 μCi/ml of platelet-rich plasma) at 37 °C for 1 h. The total loading values were obtained by lysis of equal volume of *Stx8*^−/−^ and WT unstimulated platelets in 6% glutaraldehyde with 0.1% Triton X-100, which were then transferred to scintillation fluid in 3-ml vials and CPM (counts per minute) were measured using a scintillation counter.

##### In Vitro Thrombus Formation in Whole Blood

Thrombus formation in whole blood was measured as previously described (30). Mouse blood anticoagulated with sodium citrate was collected as described above, with additional heparin (2 units/ml), and d-phenylalanyl-l-propyl-l-arginine chloromethyl ketone (40 μm) was labeled with DiOC_6_ (1 μm) and passed over immobilized collagen (50 μg/ml) or fibrinogen (100 μg/ml) (all Sigma) through a parallel plate perfusion chamber at a shear rate of 1000 s^−1^ for 3 min. Fluorescence images were captured at 4 frames/s with a BX51WI microscope (Olympus UK, Southend-on-Sea, UK) using a ×40 water dipping objective, a Rolera-XR digital camera, and QCapture software (QImaging, Surrey, BC, Canada). Coverslips were then washed with HEPES-Tyrode's buffer to remove non-adherent cells and 30 random images were collected for each experiment. Surface coverage during flow and post washing was analyzed using ImageJ.

##### In Vivo Thrombus Formation

*In vivo* thrombus formation assays were performed as previously described (30). Mice were anesthetized with an intraperitoneal injection of 100 mg/kg of ketamine and 10 mg/kg of xylazine. Vascular access was obtained by catheterization of the left external jugular vein. Platelets were labeled by intravenous administration of 100 mg/kg of Dylight 488-conjugated anti-GPIbβ antibody. Right carotid arteries were exposed using aluminum foil and 2 × 1-mm 15% ferric chloride-soaked filter paper placed on the arterial adventitia for 3 min. Time-lapse microscopy of the injury site for 20 min was performed and images processed using ImageJ. Background florescence values were measured upstream of the injury site were subtracted from thrombus-specific fluorescence and the data expressed as integrated density values. The number of embolization events was counted by reviewing the videos of each experiment 3 times (blinded).

##### Tail Bleeding Assay

Mice were anesthetized as described above. Using a scalpel, 5 mm of tail was resected from the tip, and the tail was immersed in saline (37 °C). Times from incision to cessation of bleeding were recorded.

##### Electron Microscopy

Mouse platelet samples were prepared for TEM at the University of Bristol Wolfson Bioimaging Facility, as previously described (31).

##### Statistics

Data are presented as mean ± S.E. and statistical significance was determined by two-way ANOVA with Bonferroni post-test, performed using Prism 5.0 (GraphPad Software, San Diego, CA). *p* < 0.05 was considered significant.

## RESULTS

### 

#### 

##### Identification of a Novel SNARE STX8 in Platelets

**FIGURE 1.**
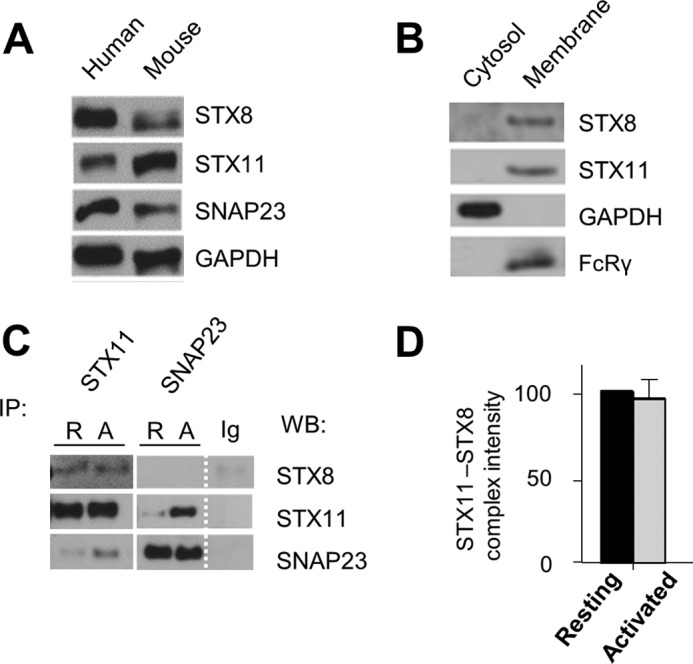
**Expression of SNARE STX8 in platelets, its localization and interactions.** *A*, resting human and mouse platelets were lysed in SDS sample buffer and proteins were separated on SDS-PAGE. The resulting blots were probed with antibodies as shown. *B,* human double washed platelets were solubilized using digitonin as described. The resulting digitonin-soluble (*cytosol*) and digitonin-insoluble (*membrane*) fractions were separated on SDS-PAGE and blotted as before and localization of STX8 was compared with localization of Fc receptor γ signaling chain (*FcR*γ, *membrane marker*), GAPDH (cytosol marker), and STX11 (membrane-associated SNARE). *C,* human resting (*R*) and activated (*A*, 1 unit/ml of thrombin) double washed platelets were lysed as described and proteins were immunoprecipitated (*IP*) with relevant antibodies. The resultant immune complexes were denatured, separated on SDS-PAGE, and blotted as before. Rabbit nonspecific Ig controls (same species as IP antibodies) were also included. *D,* the STX8-STX11 complex identified was found to be constitutive, with no significant change upon activation as quantified using ImageJ (*n* = 4). Data shown (*A–C*) are representative of at least 3 experiments, *error bars* represent S.E.

We wanted to confirm that STX8 is expressed in both mouse and human platelets. Western blotting revealed that it is present (Fig. 1*a*), alongside the other previously reported SNAREs SNAP23 and STX11. This is the first report of expression of STX8 at the protein level in platelets. To confirm its membrane localization we performed digitonin fractionation and found that it localized to the same digitonin-insoluble fraction as FcRγ and another SNARE, STX11, which confirmed membrane association of these proteins (Fig. 1*B*). Using a co-immunoprecipitation approach, we found that STX8 formed a complex with STX11, deletion of which is responsible for platelet secretion defects in FHL4 patients (15), in both resting and thrombin-activated platelets (Fig. 1*C*). That complex was not significantly enhanced in activated platelets (Fig. 1*D*). We have not found any interaction of Qb STX8 with Qbc SNAP23, consistent with the requirement of only one Qb SNARE domain for the functional SNARE complex (Fig. 1*C*). VAMP8-STX8 interaction was previously reported in the PC12 cell endosomal transport network (32), but we could not replicate that observation in platelets. This may suggest that another R SNARE is present in the complex with STX8 and STX11, consistent with the ranked redundancy of R SNAREs in platelets (16). The constitutive STX8-STX11 association is in contrast with the previously reported platelet SNARE complex, STX11-SNAP23-VAMP8, which under our solubilization conditions (as per Karim *et al.* (20)) was confirmed to be activation dependent (Fig. 1*c* and data not shown). Despite using four different STX8 antibodies in this project (Synaptic Systems polyclonal rabbit anti-STX8 (number 110-083), Santa Cruz Biotechnology monoclonal mouse anti-STX8 (48) (sc-136092), in-house rabbit polyclonal anti-STX8 antibody developed at the University of Bielefeld, and R&D Systems sheep polyclonal anti-STX8 antibody (AF5448)) we did not manage to immunoprecipitate STX8 in this project to provide the reciprocal control (data not shown).

##### Analysis of Stx8^−/−^ Mouse Platelets

Association with STX11 identified in human platelets suggested a possibility for functional importance of STX8. Therefore we obtained a global mouse knock-out model to investigate the role of STX8 in platelets.

**FIGURE 2.**
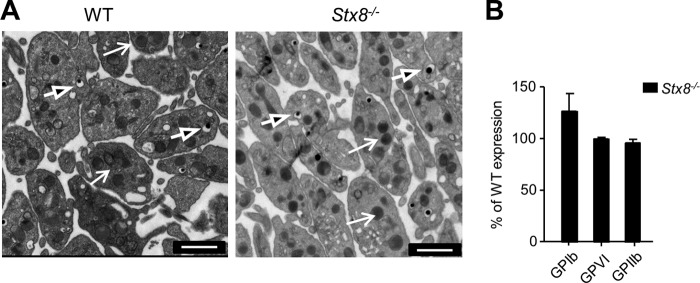
**Initial characterization of *Stx8*^−/−^ platelets.** *A,* resting platelets were prepared for TEM imaging, and images were acquired using Tecnai-12 electron microscope at ×4300 magnification. *Arrows* denote α granules, *arrowheads* denote dense granules (*scale bar*: 1 μm). Images are representative of 3 independent observations. *B,* the levels of glycoprotein expression were measured using FACS and compared with expression levels of WT platelets. There was no significant difference between the genotypes (*n* = 5, *p* > 0.05, two-way ANOVA). *Error bars* represent S.E.

*Stx8*^−/−^ mice have comparable erythrocyte, leukocyte, and platelet counts to wild-type, and platelets from these mice were normal in size (Table 1). Electron microscopy of resting *Stx8*^−/−^ platelets confirmed normal platelet morphology, with both dense and α-granules present (Fig. 2*a*). Surface expression of the α_IIb_ subunit of the fibrinogen receptor (GP_IIb_), the collagen receptor GPVI, and the GPIbα subunit of the von Willebrand factor receptor were equivalent in *Stx8*^−/−^ and WT platelets, despite GPIbα levels being more variable in *Stx8*^−/−^ (Fig. 2*B*).

**TABLE 1:** **Hematology parameters of *Stx8*^−/−^ mice** Hematology parameters were measured in whole anticoagulated blood (adjusting for the volume of anticoagulant). There was no difference in platelet count, mean platelet volume (MPV), red blood cell count (RBC), or white blood cell counts (WBC) between the genotypes (ANOVA, *p* > 0.05).

	WT			*Stx8*^−/−^			*p* value
	Mean	S.E.	*n*	Mean	S.E.	*n*	
Platelet count (×10^3^/ml)	843	31	43	833	30	46	NS*^a^*
MPV (μm^3^)	5.2	0.0	43	5.2	0.0	45	NS
WBC (×10^3^/ml)	7.6	0.5	42	6.7	0.4	45	NS
RBC (×10^3^/ml)	9.56	0.24	43	9.99	0.25	46	NS

*^a^* NS, not significant.

##### STX8 Is Specifically Required for Dense Granule Secretion, and Not α-Granule and Lysosome Secretion in Thrombin-activated Mouse Platelets

**FIGURE 3.**
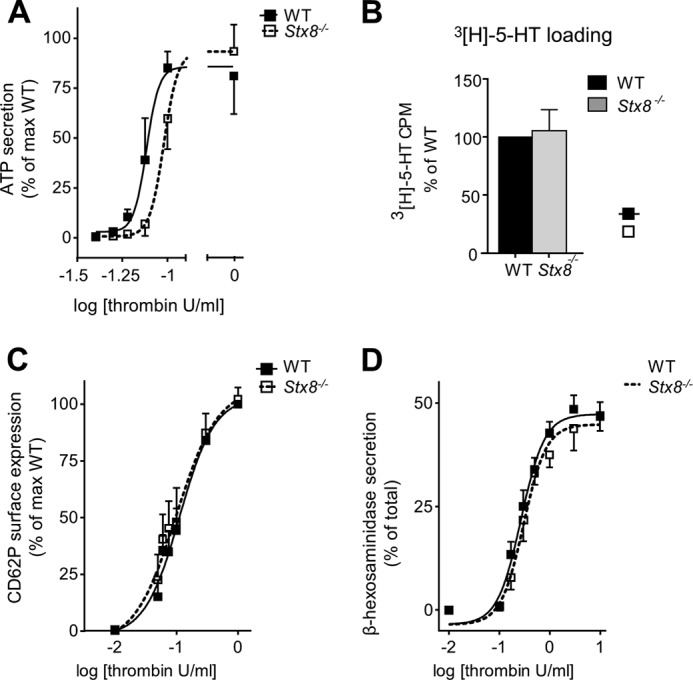
**Quantification of granule secretion in *Stx8*^−/−^ platelets.** All concentration-response data were analyzed by appropriate curve fitting in GraphPad Prism. *A,* mouse platelets were stimulated in the aggregometer as described. Dense granule secretion was measured as an increase in luminescence relative to ATP standard for each stimulation. Data were normalized to maximum ATP secretion of WT platelets on the day of experiment. There was a significant increase in [thrombin] EC_50_ in *Stx8*^−/−^ (95% CI of EC_50_: 0.085 to 0.11 units/ml) compared with WT platelets (95% CI of EC_50_: 0.068 to 0.086 units/ml) (*n* = 5, variable slope sigmoidal dose-response curve, *p* = 0.0248). *Stx8*^−/−^ and WT secretion in response to 0.06 units/ml was, respectively, 1.742 ± 0.618 and 10.379 ± 3.767% of the maximum WT secretion on the day (*n* = 4, *p* > 0.05, ns), and in response to 0.075 units/ml of thrombin: 6.927 ± 5.970 and 38.933 ± 20.969% of the maximum WT secretion, *p* = 0.0339). *B,* the total 5-HT content was measured using ^3^H-labeled 5-HT as described. There was no significant difference in 5-HT content between genotypes (*n* = 3). *C,* α-granule secretion was measured by FACS. Median fluorescent intensity (MFI) of FITC-CD62P was quantified in response to increasing concentrations of thrombin. MFI was normalized to the maximum MFI of WT platelets on the day of experiment (*n* = 4). *D,* lysosome secretion was measured by β-hexosaminidase enzyme activity in supernatants following stimulation with increasing concentrations of thrombin. Absorbance at 405 nm was measured, and values were expressed as percentage of TOTAL control for each subject (*n* = 6). There was no difference in [thrombin] EC_50_ between genotypes in α-granule or lysosome secretion (*p* > 0.05, variable slope sigmoidal dose-response curve). *Error bars* represent S.E.

Assessing ATP release from dense granules by lumi-aggregometry, we were able to show that *Stx8*^−/−^ platelets released less ATP compared with the WT, and there was a significant difference in EC_50_ values in *Stx8*^−/−^ platelets (Fig. 3*A*). The defect was more pronounced at low concentrations of agonist, and maximum secretion was comparable, suggesting no defect in granule contents was available for release. To further confirm that the defect lies in secretion rather than packaging of granules, we measured total [^3^H]5-HT loading and found no difference between genotypes (Fig. 3*B*). In contrast to dense granule secretion, there was no difference in α-granule or lysosome secretion between genotypes.

##### Stx8^−/−^ Platelets Have an Aggregation Defect Secondary to Diminished Dense Granule Secretion

**FIGURE 4.**
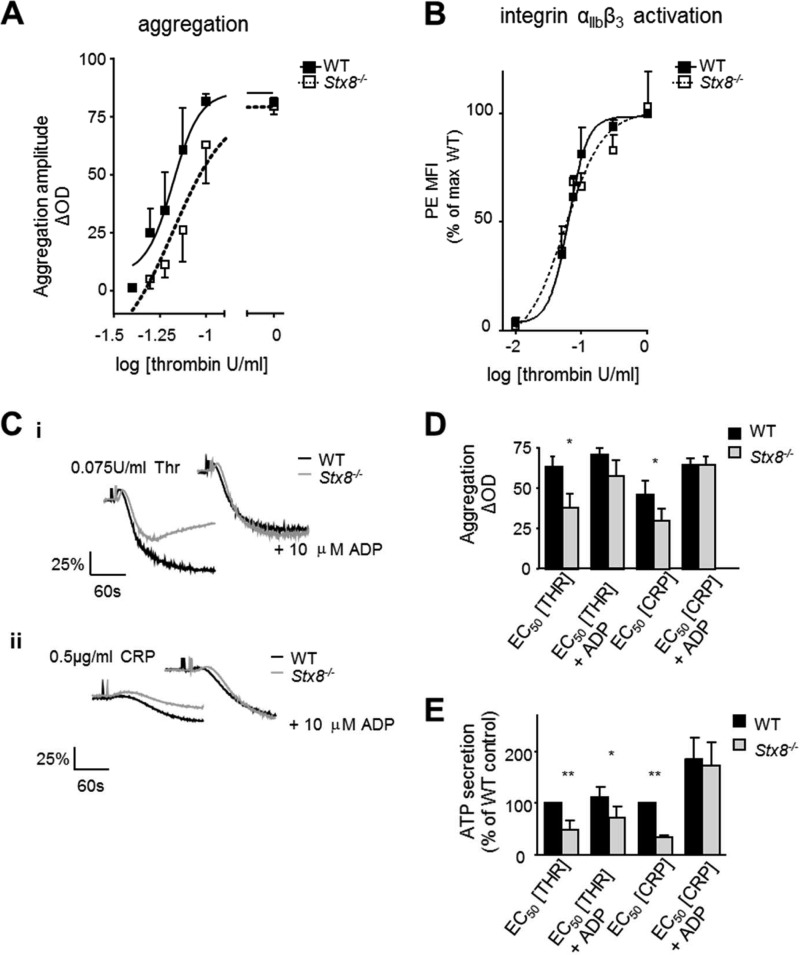
**Analysis of aggregation and ADP rescue experiments in *Stx8*^−/−^ platelets.** *A,* aggregation was measured using lumi-aggregometer and amplitude was expressed as percentage decrease in optical density of the sample at *t* = 180 s (*n* = 4). The concentration-response curve for *Stx8*^−/−^ was shifted to the right, but [thrombin] EC_50_ was not significantly different (95% CI of WT EC_50_: 0.042 to 0.090, 95% CI of *Stx8*^−/−^ EC_50_: 0.069 to 0.101, *p* > 0.05). However, the maximum aggregation amplitude was significantly reduced in *Stx8*^−/−^ at 0.075 units/ml of thrombin (26.1 ± 13.6% decrease in optical density in *Stx8*^−/−^ *versus* 60.8 ± 18.2% in WT, *p* = 0.021, two-way ANOVA with Bonferroni post-test), with aggregation at 0.05 and 0.06 units/ml also consistently reduced, but not significantly. *B,* integrin α_IIb_β_3_ activation was measured by FACS, no difference in PE-JON/A binding was observed between genotypes suggesting an alternative mechanism for the observed aggregation defect. *C,* representative aggregation traces showing the effect of co-stimulation with 10 μm exogenous ADP response to EC_50_ concentration of thrombin (*i*) and CRP (*ii*). *D,* aggregation in response to co-stimulation with ∼EC_50_ (0.05–0.075 units/ml) thrombin and 10 μm ADP was compared with EC_50_ thrombin alone (*n* ≥ 5). *Stx8*^−/−^ aggregation was significantly reduced in response to thrombin alone (37.8 ± 8.9 *versus* 63.4 ± 6.1% decrease in optical density, respectively, *p* < 0.05) and could be fully rescued with ADP (*p* > 0.05). The maximum aggregation to 1 unit/ml of thrombin was the same between genotypes (data not shown). Similarly, the effect of co-stimulation with ADP and EC_50_ CRP (0.3–0.5 μg/ml) was measured (*n* ≥ 4). There was a significant reduction in aggregation in response to ∼EC_50_ CRP in *Stx8*^−/−^ platelets (30.0 ± 7.3 *versus* 46.3 ± 8.6% in WT, *p* < 0.05) that was fully rescued with ADP (*p* > 0.05). Maximum response (to 5 μg/ml of CRP) was the same (data not shown). *E,* secretion of ATP was also measured. Again, secretion in response to EC_50_ concentrations of thrombin or CRP was significantly reduced (*p* < 0.01), whereas co-stimulation with ADP could only partially rescue secretion when thrombin was used as primary agonist (*p* < 0.05). In the case of co-stimulation with CRP, ADP enhanced ATP secretion to a much greater extent in both WT and *Stx8*^−/−^ platelets (ADP enhancement of 185.75 ± 42.55% of CRP alone, compared with 111.30 ± 20.66% of thrombin alone). *Error bars* represent S.E.

We also observed a defect in platelet aggregation that followed a similar pattern to ATP secretion (Fig. 4*A*). We suspected that the defect results from insufficient ADP-mediated positive feedback at low agonist concentrations. In contrast, integrin α_IIb_β_3_ activation was normal when *Stx8*^−/−^ platelets were stimulated in 10-fold diluted suspension (2 × 10^7^/ml), suggesting that there was no intrinsic defect in the ability of the integrin to be activated (Fig. 4*b*). To confirm that the aggregation defect was secondary to dense granule secretion we repeated the aggregations in the presence of exogenous ADP, which is normally released from dense granules upon stimulation and can synergistically propagate aggregation and further dense granule secretion. When platelets were co-stimulated with low concentrations of agonists, thrombin (0.05–0.075 unit/ml) (Fig 4ci) or CRP-XL (0.3–0.5 μg/ml) (Fig. 4*C*, *ii*) and 10 μm ADP, the significant defect in aggregation could be fully rescued in *Stx8*^−/−^ platelets (Fig. 4*D*). Dense granule secretion, however, remained significantly reduced in *Stx8*^−/−^ platelets after thrombin-ADP co-stimulation (Fig. 4*e*). CRP-ADP co-stimulation resulted in much greater potentiation of secretion (∼200% compared with ∼25% increase in thrombin-stimulated platelets), masking any potential difference still present in *Stx8*^−/−^ (Fig. 4*E*).

##### STX8 Deletion Leads to Down-regulation of Another SNARE VTI1B, Which, However, Is Not Involved in Secretion

**FIGURE 5.**
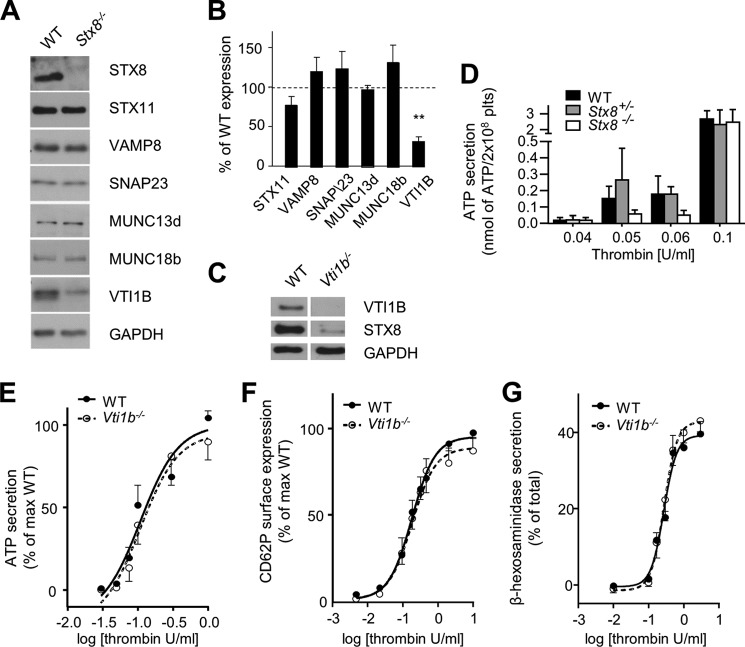
**Analysis of secretion in *Vti1b*^−/−^ platelets.** *A,* expression levels of other known SNAREs and SNARE-associated proteins were measured in *Stx8*^−/−^ platelets to ensure the defect results from Stx8 deletion. Levels of STX11, VAMP8, SNAP23, and MUNC13d and 18b were comparable between the genotypes (image representative of at least 3 independent experiments). *B,* a significant decrease in expression of Vti1b in *Stx8*^−/−^ platelets was observed (31.13 ± 5.85% of WT expression, *p* < 0.01). *C,* similarly, Stx8 was also down-regulated in *Vti1b*^−/−^ platelets. *D,* to confirm that only complete ablation of Stx8 leads to defective dense granule secretion, we analyzed ATP secretion from *Stx8*^+/−^ platelets and found no defect, unlike in *Stx8*^−/−^ platelets (*n* > 3, difference not significant but consistent for WT *versus Stx8*^−/−^, two-way ANOVA). *E–G,* to further confirm that deletion of Stx8 and not the associated down-regulation of Vti1b was responsible for defect in dense granule secretion observed, we measured secretion from dense (*E*) and α-granules (*F*) and lysosomes (*G*) in *Vti1b*^−/−^ platelets. No difference was observed in any of the secretion events in *Vti1b*^−/−^ platelets (*n* = 6). *Error bars* represent S.E.

To ensure that the defects observed were truly due to STX8 knock-out, we analyzed expression levels of SNAREs and Munc/SEC family members thought to be important in platelet secretion (Fig. 5*A*). No difference in expression was found (Fig. 5*B*). We did, however, find that levels of another primarily endosomal SNARE, VTI1B, known to form complexes with STX8 in other cells, were significantly reduced (the converse has also been reported previously in *Vti1b*^−/−^ hepatocytes (27), and confirmed here, Fig. 5*C*).

The defect in dense granule secretion was not present in *Vti1b*^−/−^, suggesting that this SNARE, despite being expressed in platelets, was not involved in the same secretion events as STX8 (Fig. 5, *E–G*). Similarly, dense granule secretion in *Stx8*^+/−^ platelets was not changed, further confirming that complete ablation of STX8 is required for the defect to occur (Fig. 5*D*).

##### Stx8^−/−^ Platelets Show Normal Thrombus Formation under Flow in Vitro and in Vivo and Normal Tail Bleeding Times, but Have Defective Thrombus Stability in Vivo

**FIGURE 6.**
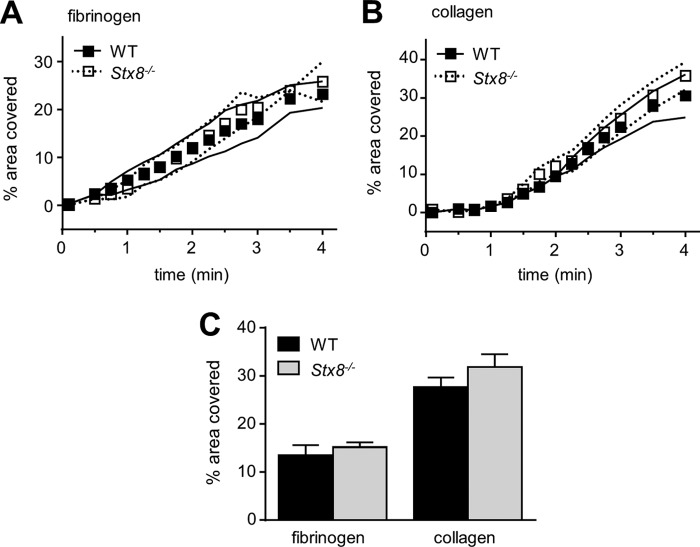
**Analysis of *in vitro* thrombus formation under shear in *Stx8*^−/−^ whole blood.** Anti-coagulated whole blood was flowed over immobilized collagen (50 μg/ml) or fibrinogen (100 μg/ml) through a parallel plate perfusion chamber at a shear rate of 1000 s^−1^ for 4 min. *A* and *B,* the chamber was constantly imaged over the time of experiment and the percentage coverage at each time point was acquired using QCapture software. The change in coverage represents mean ± S.E. (*n* ≥ 4). *C,* at the end of each experiment, chambers were washed for 3 min with buffer to remove non-adherent platelets and erythrocytes, and 30 random images of the whole coverslip were taken. The surface area covered by thrombi was analyzed using ImageJ (*n* ≥ 4). There was no difference in either the rate of adhesion or the size of thrombi in *Stx8*^−/−^, either on collagen (GPVI-mediated adhesion) or on fibrinogen (α_IIb_β_3_-mediated adhesion). *Error bars* represent S.E.

We observed thrombus formation on two surfaces, collagen and fibrinogen, under a shear stress of 1000 s^−1^. We found that there was no difference either in the rates of adhesion (Fig. 6, *A* and *B*) or in final coverage after 4 min of flow between *Stx8*^−/−^ and WT platelets (Fig. 6*C*).

**FIGURE 7.**
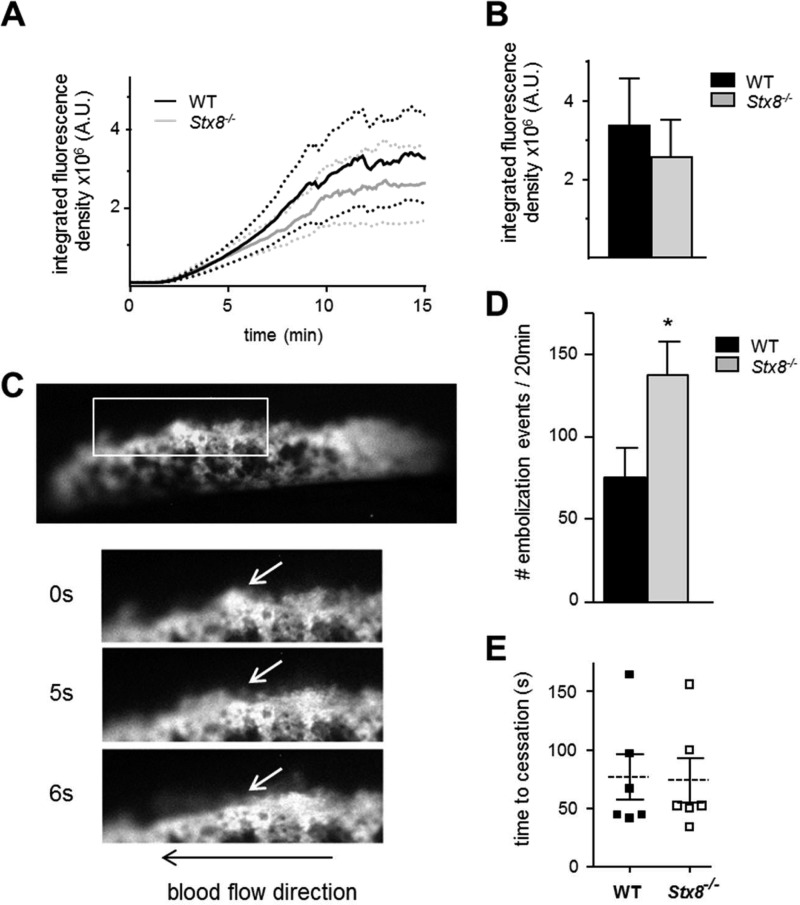
***In vivo* thrombus formation and embolization of analysis in *Stx8*^−/−^ and WT animals.** Carotid artery damage was achieved by treatment with FeCl_3_ as described. Fluorescently labeled platelets adhering at the site of injury could then be imaged continuously. *A,* the rate of platelet adhesion was slower in *Stx8*^−/−^, but the difference was not significant (adhesion rate shown as mean ± S.E.). *B,* the final thrombi size (expressed as IFD of the field of view) at the end of a 20-min experiment was also reduced in *Stx8*^−/−^ animals but the difference was not significant. The videos were then reviewed again, focusing on the number of embolization events as shown in *C*. An embolus, seen here as a small fragment of the shell of the growing thrombus (*arrow* at 0 s) becomes dislodged and initially “rolls” with the direction of blood flow (at 5 s) to eventually become detached from the thrombus and carried away by the blood flow (6 s). *D,* when counted throughout the duration of the experiment, the number of embolization events was significantly increased in the *Stx8*^−/−^ (137.3 ± 20.12) compared with WT animals (75.46 ± 17.71) (*n* = 8, *p* = 0.037, *t* test). *E,* mean time to cessation of bleeding in tail bleeding assay was the same in *Stx8*^−/−^ and WT animals (*t* < 180 s, *n* = 6). *Error bars* represent S.E.

Similarly, both the average adhesion rate at the site of FeCl_3_ injury and the final size of thrombus were only slightly reduced in *Stx8*^−/−^ animals, and the defects were not significant (Fig. 7, *A* and *B*). Interestingly, however, when the videos were analyzed for numbers of embolization events (Fig. 7*C* and supplemental Videos S1 and S2) occurring during the experiment, we found that thrombi formed in *Stx8*^−/−^ animals were less stable and significantly more likely to embolize (Fig. 7*D*).

The finding that thrombus stability was altered in *Stx8*^−/−^ mice prompted assessment of their hemostatic potential. Tail bleeding times were normal in *Stx8*^−/−^ mice (Fig. 7*E*), suggesting that Stx8 deletion despite affecting thrombosis, does not lead to hemostatic abnormalities.

## DISCUSSION

Although functional redundancy of mammalian (33), and indeed platelet (16) SNAREs was demonstrated previously, the one size fits all model remains generally accepted in platelets, where the STX11-SNAP23-VAMP8 complex is proposed as the predominant SNARE complex regulating platelet secretion. Until now, there was no other candidate Qbc SNARE(s) in platelets apart from SNAP23. However, the evidence in support of SNAP23 remains incomplete. The initial studies suggesting its role used the permeabilized platelet model, where Fab antibody fragments targeting SNAP23 were introduced into platelets before stimulation with calcium (17, 18, 34). Chen *et al.* and Lemons *et al.* (17–19) found a significant, but not complete ablation of all granule secretion using this assay. In the same model, STX2 and -4 were shown to be important SNAREs for all secretion events (17–19). Recently it was shown that the antibodies used were in fact nonspecific and also blocked STX11 (15), now known to be responsible for the secretion defect in FHL-4. In light of the lack of other conclusive evidence, investigating SNAREs that could “help” SNAP23 in regulating secretion was a promising direction.

In this study we show evidence for expression of the SNARE STX8, previously unstudied in platelets. Our data suggest that it could, possibly in complexes with “confirmed” SNARE STX11 and as yet unidentified Qb and R SNAREs, contribute to the regulation of dense granule secretion.

STX8 was initially found in the endosomal SNARE complex (14) but no functional data in any cell type on *Stx8*^−/−^ knock-out mice is known to date. It was found to play a role in cystic fibrosis transmembrane conductance regulator trafficking in epithelial cells (35, 36), and Golgi-to-plasma membrane trafficking of TrkA receptor in neurons (37) supporting its role in endosomal trafficking. STX8 was, however, also found on the plasma membrane of PC12 cells (38) suggesting that its di-leucine-based motif may differentially function in both exocytotic and endocytotic pathways. Recently, STX8 was also found to localize with lytic granules in cytotoxic T leukocytes, suggesting a role in secretion in cells of hematopoietic lineage (39).

Platelet dense granules are lysosome-like organelles that originate from multivesicular bodies in megakaryocytes (40) and STX8 has been implicated in endosome-to-lysosome transport, first in the NRK cell line (41), and also in macrophages (42). It was possible that a STX8-containing SNARE complex is involved in endosomal transport steps in megakaryocytes but we found no apparent difference in platelet or granule morphology. The 5-HT content of dense granules also appeared to be equal. Therefore STX8 is unlikely to play a role in granule formation.

Karim *et al.* (20) recently showed that the VAMP8-SNAP23-STX11 complex associates following activation with thrombin. In contrast, the novel STX8-STX11 interaction appeared to be constitutive. If a pre-assembled complex was present in addition to an activation-dependent SNAP23-VAMP8-STX11 complex, it could indicate a subpopulation of dense granules that could be released upon weaker stimulation. One possibility would be the granules that are pre-docked to the target membrane. We did not, however, find STX8 in complexes with VAMP8 suggesting that either a different v-SNARE is involved, or the lysis conditions did not preserve that interaction. Alternatively, the increased affinity of the complex could be due to differences in accessory secretion regulators interacting with the complex, such as the MUNC family, small GTPases or others (43), but the role of many of those in platelets remains to be elucidated.

The defect in dense granule secretion we observe under stimulation with intermediate concentrations of agonists in *Stx8*^−/−^ platelets could support this general model. A recent study showed that release of distinct α-granule cargo molecules is kinetically heterogeneous and dependent on agonist (type, rather than concentration) but no mechanistic data exists to explain this (44). Dense granule secretion is even less well studied and is thought to occur very rapidly, but in view of the range of functions that dense granule cargo small molecules play in the vascular environment, it is likely that a level of spatial and temporal regulation is required as well (45). STX8 may contribute to that regulation. STX11 is an unusual SNARE in that it does not contain a traditional transmembrane domain (46). It is thought that association with MUNC18b (47) together with putative post-translational modifications (46) are required for its membrane anchoring and stabilization, however, association with a transmembrane syntaxin such as STX8 may also contribute to this stabilization. This could lead to differential complex dynamics and provide an additional mechanism for dense granule secretion in response to low agonist stimulation.

Rather than any up-regulation, we observed a reduction in the expression levels of VTI1B in *Stx8*^−/−^ platelets with the expression of other known SNAREs and MUNCs not affected. Lack of a comparable dense granule secretion defect in *Vti1b*^−/−^ platelets excludes it as an essential binding partner for STX8 in this secretion event, despite previous reports of VTI1B-STX8 pairing in membrane trafficking events in other cell types (14, 27, 41, 42).

The defect in dense granule secretion and aggregation in *Stx8*^−/−^ platelets did not translate to a defect in thrombus formation in whole blood under shear. However, because we did not see a defect in secretion at high levels of stimulation either with thrombin or CRP, this may suggest that the role of STX8 is very specific to an intermediate level of stimulation. The level of stimulation in the *in vitro* thrombus formation assay used would activate positive feedback cascades and lead to an increase in dense granule secretion that would most likely disguise any defects in primary secretion.

Similarly, in the ferric chloride injury model, damage to the vessel wall results in exposure of extracellular matrix far exceeding the concentrations applied in an aggregation assay and correspondingly, the total size of thrombus was only slightly reduced in *Stx8*^−/−^ animals. However, importantly, it was clear that *Stx8*^−/−^ thrombi embolized more readily, and that the difference was significant. This could align with the proposed “core and shell” structure to thrombi (48), because the core consists of more activated, P-selectin expressing platelets that would not be affected in the *Stx8*^−/−^ mice (because P-selectin expression was normal in *Stx8*^−/−^). The “shell” consists of “less” activated, loosely attached platelets and its stability depends on P2Y_12_ signaling. Therefore the shell would be more sensitive to changes in ADP signal intensity, such as is present in the *Stx8*^−/−^ mouse where there is decreased dense granule secretion. No difference in tail bleeding time between genotypes further suggests that STX8 is more important in thrombosis than in the hemostatic response.

The observation that STX8-mediated dense granule secretion contributes to thrombus stability may have implications in our search for selective anti-thrombotic agents that spare primary hemostasis (49). This analysis emphasizes that it is worth considering alternative end points for analysis of *in vivo* gene knock-out phenotypes presenting with less profound functional defects *in vitro*. In the clinical setting, shedding of emboli from the site of initial thrombus formation may give rise to occlusive thrombus formation downstream in the vasculature and lead to ischemic events. Whereas the effect of shear on adhesion and aggregation in the vasculature was recently reviewed elsewhere (50), the secretion underlying thrombus stabilization under flow remains to be elucidated.

In summary, we have shown that a novel SNARE protein, interacting with the previously reported important SNARE STX11 contributes selectively to dense granule secretion. This translates into a relevant increase in thrombus stability *in vivo*. Further studies are needed to understand the importance of this in the full range of physiological and pathological processes mediated by platelet dense granule secretion.

## Supplementary Material

Supplemental Data

## Footnotes

2 The abbreviations used are:

STX: syntaxin
ANOVA: analysis of variance
5-HT: 5-hydroxytryptamine
CRP: collagen-related peptide
GP: glycoprotein
PE: phycoerythrin.
