# Supplementary material for: Syntaxin 8 Regulates Platelet Dense Granule Secretion, Aggregation, and Thrombus Stability
Source: J Biol Chem. 2014 Nov 17;290(3):1536–45. doi: 10.1074/jbc.M114.602615 (PMC4340400; doi:10.1074/jbc.M114.602615)
Supplement: Supplemental Data [file supp_290_3_1536__index.html]

Syntaxin 8 Regulates Platelet Dense Granule Secretion, Aggregation and Thrombus Stability — Syntaxin 8 Regulates Platelet Dense Granule Secretion, Aggregation, and Thrombus Stability — Syntaxin 8 in Platelet Secretion and Function — Supplemental Data 

# Syntaxin 8 Regulates Platelet Dense Granule Secretion, Aggregation, and Thrombus Stability

## Supplemental Data

**Files in this Data Supplement:**

- Supplemental video 1 (.avi, 2.7 MB) - In vivo thrombus formation - WT, t=10-11.5min
- Supplemental Video 2 (.avi, 5.1 MB) - In vivo thrombus formation - STX8 KO, t=10-11.5min
